# Computed tomography and structured light imaging guided orthopedic navigation puncture system: effective reduction of intraoperative image drift and mismatch

**DOI:** 10.3389/fsurg.2024.1476245

**Published:** 2024-10-10

**Authors:** Zaopeng He, Guanghua Xu, Guodong Zhang, Zeyu Wang, Jingsong Sun, Wei Li, Dongbo Liu, Yibin Tian, Wenhua Huang, Daozhang Cai

**Affiliations:** ^1^ The Third Affiliated Hospital and Third School of Clinical Medicine, Southern Medical University, Guangzhou, China; ^2^ Lecong Hospital of Shunde, Foshan, China; ^3^ Guangdong Engineering Research Center for Translation of Medical 3D Printing Application, Guangdong Provincial Key Laboratory of Medical Biomechanics, National Key Discipline of Human Anatomy and School of Basic Medical Sciences, Southern Medical University, Guangzhou, China; ^4^ Department of Orthopedics, Affiliated Hospital of Putian University, Putian, China; ^5^ School of Basic Medical Sciences, Yanbian University, Yanbian, China; ^6^ College of Mechatronics and Control Engineering, Shenzhen University, Shenzhen, China; ^7^ Orthopedic Hospital of Guangdong Province, Academy of Orthopedics Guangdong Province, Guangzhou, China

**Keywords:** computed tomography, structured light imaging, surgical navigation system, orthopedic puncture surgery, image registration

## Abstract

**Background:**

Image-guided surgical navigation systems are widely regarded as the benchmark for computer-assisted surgical robotic platforms, yet a persistent challenge remains in addressing intraoperative image drift and mismatch. It can significantly impact the accuracy and precision of surgical procedures. Therefore, further research and development are necessary to mitigate this issue and enhance the overall performance of these advanced surgical platforms.

**Objective:**

The primary objective is to improve the precision of image guided puncture navigation systems by developing a computed tomography (CT) and structured light imaging (SLI) based navigation system. Furthermore, we also aim to quantifying and visualize intraoperative image drift and mismatch in real time and provide feedback to surgeons, ensuring that surgical procedures are executed with accuracy and reliability.

**Methods:**

A CT-SLI guided orthopedic navigation puncture system was developed. Polymer bandages are employed to pressurize, plasticize, immobilize and toughen the surface of a specimen for surgical operations. Preoperative CT images of the specimen are acquired, a 3D navigation map is reconstructed and a puncture path planned accordingly. During surgery, an SLI module captures and reconstructs the 3D surfaces of both the specimen and a guiding tube for the puncture needle. The SLI reconstructed 3D surface of the specimen is matched to the CT navigation map via two-step point cloud registrations, while the SLI reconstructed 3D surface of the guiding tube is fitted by a cylindrical model, which is in turn aligned with the planned puncture path. The proposed system has been tested and evaluated using 20 formalin-soaked lower limb cadaver specimens preserved at a local hospital.

**Results:**

The proposed method achieved image registration RMS errors of 0.576 ± 0.146 mm and 0.407 ± 0.234 mm between preoperative CT and intraoperative SLI surface models and between preoperative and postoperative CT surface models. In addition, preoperative and postoperative specimen surface and skeletal drifts were 0.033 ± 0.272 mm and 0.235 ± 0.197 mm respectively.

**Conclusion:**

The results indicate that the proposed method is effective in reducing intraoperative image drift and mismatch. The system also visualizes intraoperative image drift and mismatch, and provides real time visual feedback to surgeons.

## Graphical Abstract

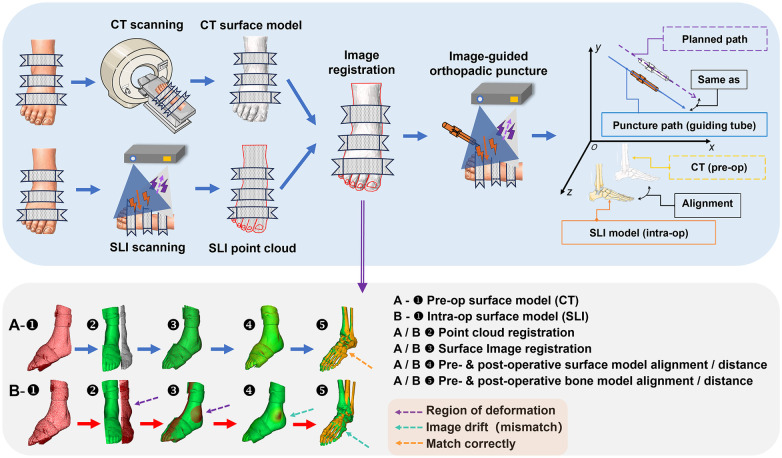

## Highlights

• A three-stage surface fixation procedure (plasticization, curing and hardening) was utilized to fix specimen to reduce intraoperative surface deformation;
• High-precision dynamic Structured Light Imaging (SLI) was developed to capture the surface profile of a long segment of the of specimen as well as a guiding tube for image-guided puncture surgery in real-time;
• The SLI surface is aligned to preoperative CT model using coarse-to-fine point cloud registration with an computationally efficient Iterative Closest Point (ICP) algorithm; guiding tube point cloud is fitted as a cylinder by RANSAC algorithm to provide real-time feedback and guide puncture by aligning with planned path from CT;
• The proposed CT and SLI image-guided puncture method has been evaluated using 20 formalin-soaked lower limb cadaver specimens, and it is effective in reducing intraoperative image drift and mismatch.

## 1 Introduction

Image-guided surgical navigation systems are widely regarded as the benchmark for computer-assisted surgical robotic platforms, which are used for minimally invasive surgery operations, especially in the fields of orthopedics (1) and neurosurgery (2). The systems can accurately locate surgical targets and minimize surgical trauma and postoperative infections, thereby improving the overall surgery success rates. Despite the relatively high mechanical precision of navigation devices produced by various orthopedic surgical navigation system manufacturers, there are some unresolved issues, such as the occurrence of intraoperative image drift and mismatch (3).

Intraoperative image drift and mismatch refer to any differences in the relative positions of pixels between images during surgical operations, which can be caused by alterations in the surface morphology or internal structure of the human body. These changes can significantly impact the 3D models and point cloud images, making it crucial for surgical navigation systems to maintain a high level of precision and robustness for the optimal surgical outcomes. The accuracy of the spatial mapping between the preoperative images (virtual space) and the intraoperative images (real space) is paramount, as it directly determines the success or failure of the surgical procedure (4).

The precision of surgical navigation systems is multifaceted and influenced by a range of factors, such as the quality of image acquisition and the precision of image registration. The tracking accuracy of surgical instruments also plays an important role, as it ensures that the system can accurately track and guide the instruments during surgery. The positioning and mode of the reference frame, and the patient's position and respiratory patterns can introduce variability, necessitating the need for adaptive tracking mechanisms. Human factors, both preoperative and intraoperative, including surgeon skill, fatigue, and other human error sources, all have the potential to introduce inaccuracies into the navigation system (5, 6). These factors may be intertwined and lead to cumulative errors (7). Addressing these factors through robust system design, continuous calibration, and surgeon training, is crucial for enhancing the precision of surgical navigation systems and ensuring patient safety.

The limited field of view in orthopedic surgeries often poses a significant challenge in achieving optimal outcomes through traditional surgical methods. Among the critical factors that impact surgical precision are pre-operative Computed Tomography (CT) scanning, intraoperative image registration, and the precision of surgical navigation systems. For example, spinal surgical operations are particularly challenging due to the potential for changes in vertebral position and morphology, which can hinder the localization of key anatomical features. The phenomenon of spatial mapping misalignment of surgical instruments is another concern. This misalignment can lead to impaired judgment for the surgeon, potentially resulting in incorrect instrument insertion (8). To address these challenges, it is important to have advanced surgical navigation systems that can provide accurate intraoperative imaging and robust instrument tracking, with real-time feedback. By optimizing these parameters, surgeons can overcome the limitations of the limited field of view and enhance the precision of orthopedic surgeries, ultimately leading to improved patient outcomes.

Currently there are some surgical navigation systems to reduce intraoperative image drift and mismatch. For instance, TINAVI Orthopaedic Robot (TINAVI, Inc., China) employs a restraining belt to limit torso displacement (9). Similarly, Mazor Robotic (Medtronic, Inc., USA) adopts pins fixation of the iliac bone (10). Sinnovation and Remebot are two additional examples of such systems. They use leksell frames in addition to screws to fix the skull, preventing the head from motor displacement (11). Augmented reality (AR) technology can superimpose virtual images onto the patient's surgical site and use translation and/or rotation to fix any mismatches. Thus, AR can enhance navigation safety and compensate for navigation errors (12). There have also been studies on multimodal image combination or preoperative dynamic updating of 3D databases to detect soft tissue drift in the brain for improved precision (13). However, the compensation results are based on statistical models, not intraoperative real-time dynamic image reconstruction results.

Some studies have reported that the intraoperative and preoperative surface images generated by different imaging devices result in misalignment and intraoperative image mismatch during surgery (14). 7D surgery Inc (Canada) incorporates LED light beads and a near infrared binocular stereo camera for surgical navigation. Intraoperative surface imaging and preoperative 3D modeling provided by the stereo camera and CT or MRI are leveraged. And ICP registration is used for image alignment (15, 16). The integration of structured light and near-infrared binocular cameras by Sinovation Inc. (China) enables the scanning of the head and face and instrument track, as well as surface alignment with preoperative CT/MRI. This technology is commonly employed in various medical procedures, such as brain hemorrhage drainage, deep brain electrical stimulation, electrode implantation, and puncture operations (17, 18). The binocular navigation systems from Polaris Spectra and Polaris Vicra (Northern Digital Inc., Canada) provide a precision of approximately 0.25 mm in their respective working volumes (3). They use a dynamic reference frame for adjacent bone localization and do not provide surgical site bone localization. The device is also affected by its own stability, tracking algorithm, working distance and other factors, and fails to fully respond to the real-time dynamic changes of the bones in the soft tissues (7, 19). Their Aurora system has an precision of 0.48 mm and a directional precision of 0.30° (3). Ascension Technology Corp. (Vermont, USA) developed an electromagnetic navigation system with a positioning precision of 1.4 mm, a directional precision of 0.5°, and an update frequency of 80 Hz (20). However, this device is commonly used in cavity organ navigation; other organ surgical navigation applications are less common, and sensor installation is difficult (21, 22). The device is susceptible to ferrous metal instruments, conductive materials, and distance, resulting in positioning errors. In addition, electromagnetic navigation equipment has its own inherent errors; factors such as magnetic field aberrations, external currents and inhomogeneous media affect its precision (3).

To overcome the issues in the above surgical navigation systems and to improve surgical precision, we have developed a CT and Structured Light Imaging (SLI) guided orthopedic navigation puncture system in combination with three-stage surface fixation and a guiding tube to reduce intraoperative image drift and mismatch, and provide real-time feedback to surgeons. Polymer bandages are employed to pressurize, plasticize, immobilize and toughen the surface of a specimen for surgical operations. Preoperative CT images of the specimen are acquired, a 3D navigation map is reconstructed and a puncture path planned accordingly. During surgery, an SLI module captures and reconstructs the 3D surfaces of both the specimen and a guiding tube for the puncture needle simultaneously. The SLI reconstructed 3D surface of the specimen is matched to the CT navigation map via coarse-to-fine point cloud registrations, while the SLI reconstructed 3D surface of the guiding tube is fitted by a cylindrical model, which is in turn aligned with the planned puncture path to guide the surgery. There are multiple objectives of such a design. First, using the SLI instead of the CT during surgery reduces patients’ exposure to radiations, as well as lowers the cost of surgical operations significantly as the SLI cost is a small fraction of the CT cost. Second, the SLI offer faster and higher precision surface 3D information of the specimen than the CT, providing real-time feedback to the surgical equipment and the surgeon which is not feasible by CT. Third, the same SLI module can also image the metal surgical instrument which the CT cannot, providing instrument tracking capability. Last but not the least, the SLI offers higher precision, higher resolution and more robust 3D output than the aforementioned near-infrared stereo systems that track limited number of light sources or reflective markers. The proposed system has been tested and evaluated using 20 formalin-soaked lower limb cadaver specimens preserved at a local hospital.

## 2 Materials and methods

### 2.1 Specimens

The study was approved in advance by the ethics committee of Le Cong Hospital in the Shunde District of Foshan City, Guangdong, China, and followed the Declaration of Helsinki. A total of 20 formalin-soaked lower limb cadaveric specimens preserved at the hospital were used for the current study.

### 2.2 Procedures for orthopedic navigation puncture experiments

As illustrated in the graphical abstract, the operation procedures of the proposed CT and SLI guided orthopedic navigation puncture system are as follows: (1) rigid plasticization and fixation of specimen; (2) preoperative CT scans to acquire a 3D navigation map; (3) intraoperative SLI scanning and 3D reconstruction, and point cloud registration between the navigation map and intraoperative SLI acquired surface in real-time; (4) tracking of the puncture needle during surgery by fitting a cylindrical model to the SLI acquired surface of the guiding tube; and (5) postoperative CT scans to verify the outcomes of puncture to evaluate intraoperative image drift and mismatch.

#### 2.2.1 Three-stage surface fixation of specimens

**Figure 1:**
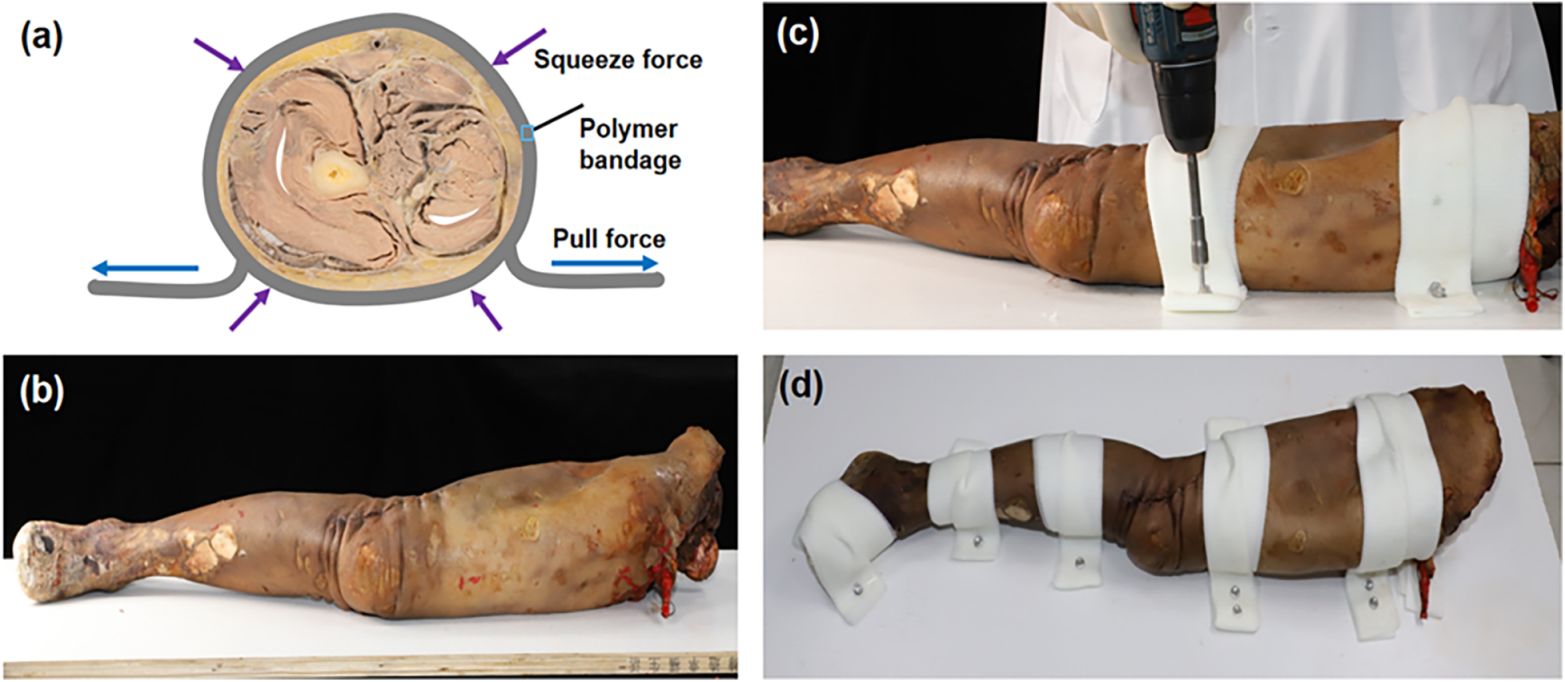
Illustration of the lower limb specimen TSSF strapping process. **(a)** Schematic of spiral pressurization of cadaveric specimen with polymer bandages; **(b)** A lower limb specimen and the immobilization board; **(c)** Polymer bandage immobilization with screws; **(d)** Lower limb specimen after the full TSSF procedure.

To reduce intraoperative image drift and mismatch, lower extremity specimens underwent binding, pressurizing, plasticizing, toughening, and screw-fixing with polymer bandages (Jingyi Cast, Yangzhou, China), hereafter referred to as Three-Stage Surface Fixation (TSSF) (Figure 1), where three-stage refers to plasticization, curing and hardening. The lower limb specimen was placed on a wooden board (size: 110 cm × 45 cm) on the inner side. The polymer bandages were folded in half to form a 1 cm folded bandage, placed in water for about 3 s, then fished out and squeezed dry. After the specimen was wrapped by the polymer bandages, it was placed in the central region of the board. The left and right ends of the polymer bandage were gently pulled to make the specimen surface appear slightly tight. After the polymer bandage was completely plasticized, both ends of the bandage are fixed to the board using screws. Equation 1 approximates the polymer bandage strapping length for the lower extremity specimen.

(1) L=2*D+10

where D is the diameter of the specimen at the location to be fixed, and the unit is in centimeter.

#### 2.2.2 3D navigation maps from CT

**Figure 2:**
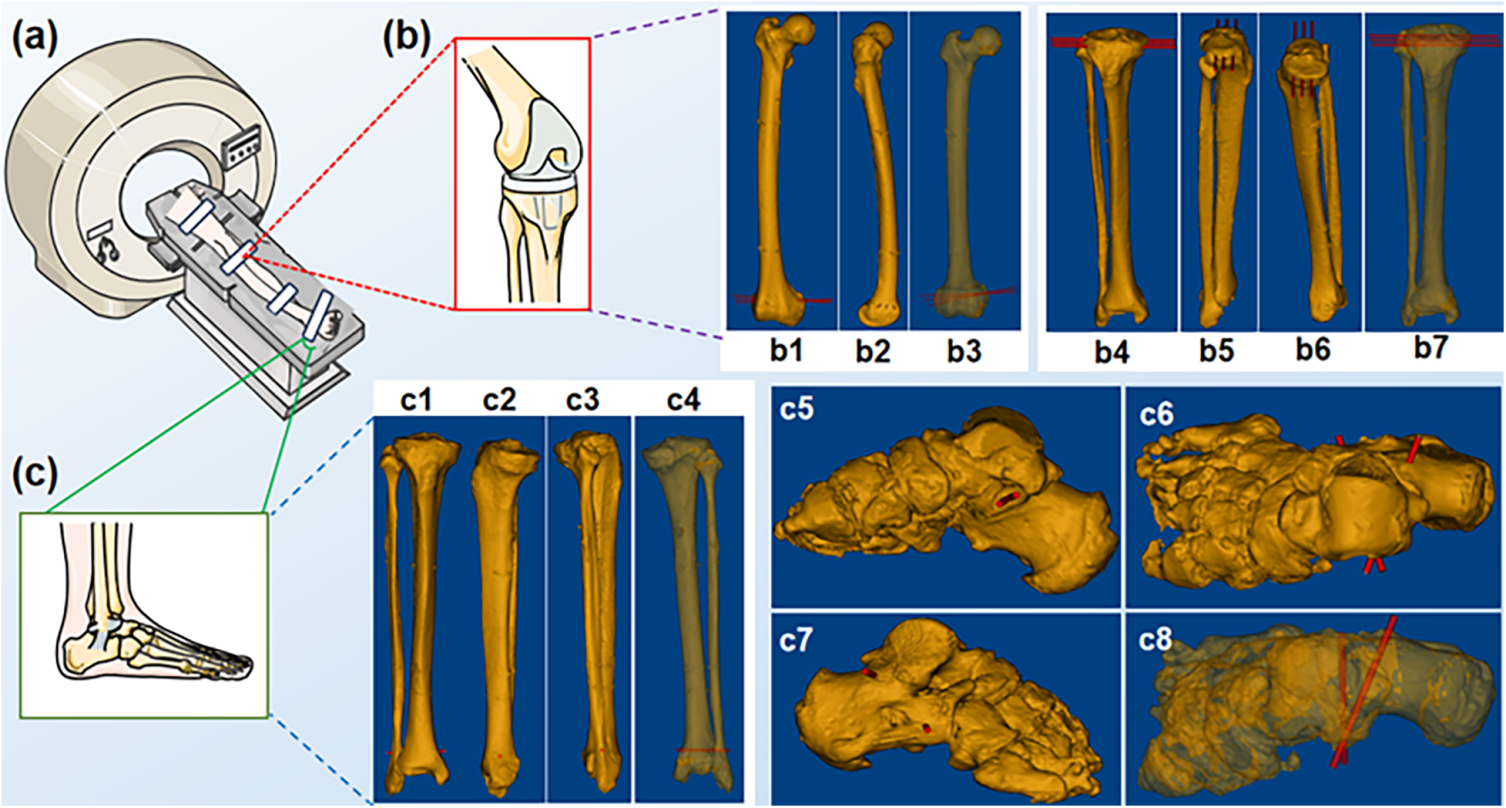
Spiral CT scans of the specimen. **(a)** Schematic of a spiral CT scan; **(b)** Knee region; (b1-b7) distal femur and tibial plateau puncture sites; **(c)** Foot region; (c1-c8) distal tibiofibular and sustentaculum tali puncture sites.

The fixed lower limb specimen was scanned by spiral CT (INGENUITY CORE 128 CT, Philips, Netherlands) using the soft tissue algorithm, with the following parameters: tube voltage of 120 KV, layer thickness of 0.675 mm, and field of view of 180 mm. DICOM images were transferred to the navigation system workstation. Then the specimen surface and the skeleton 3D models were obtained (Figure 2). This was achieved by utilizing the threshold segmentation (body surface -650HU∼Maximum; skeleton 125HU-Maximum), mask editing, region growing, contour line fitting, and 3D model reconstruction. The STL files of the reconstructed 3D models were imported into the navigation software.

#### 2.2.3 Intraoperative structured light imaging

In the intraoperative stage, a dynamic SLI 3D camera (MEGA PHASE, Shanghai, China) was employed. Structured light patterns were generated by a Digital Mirror Device (DMD) (Texas Instrument, Texas, USA) and projected onto the scene, and a CMOS sensor captured the corresponding images. A 3D profile of the scene surface was reconstructed by a triangulation method (23). The camera was fixed on top of a post at the height of 2 m, with spatial resolution of 3 megapixels. Its working distances were set to 0.9–1.5 m, and field of view to 800 mm * 605.8 mm at 1.5 m. The camera was used to scan both the lower limb specimen and the guiding tube for the puncture needle. Point clouds of a long segment of the specimen surface as well as the guiding tube were obtained.

#### 2.2.4 Specimen surface point cloud registration and alignment

**Figure 3:**
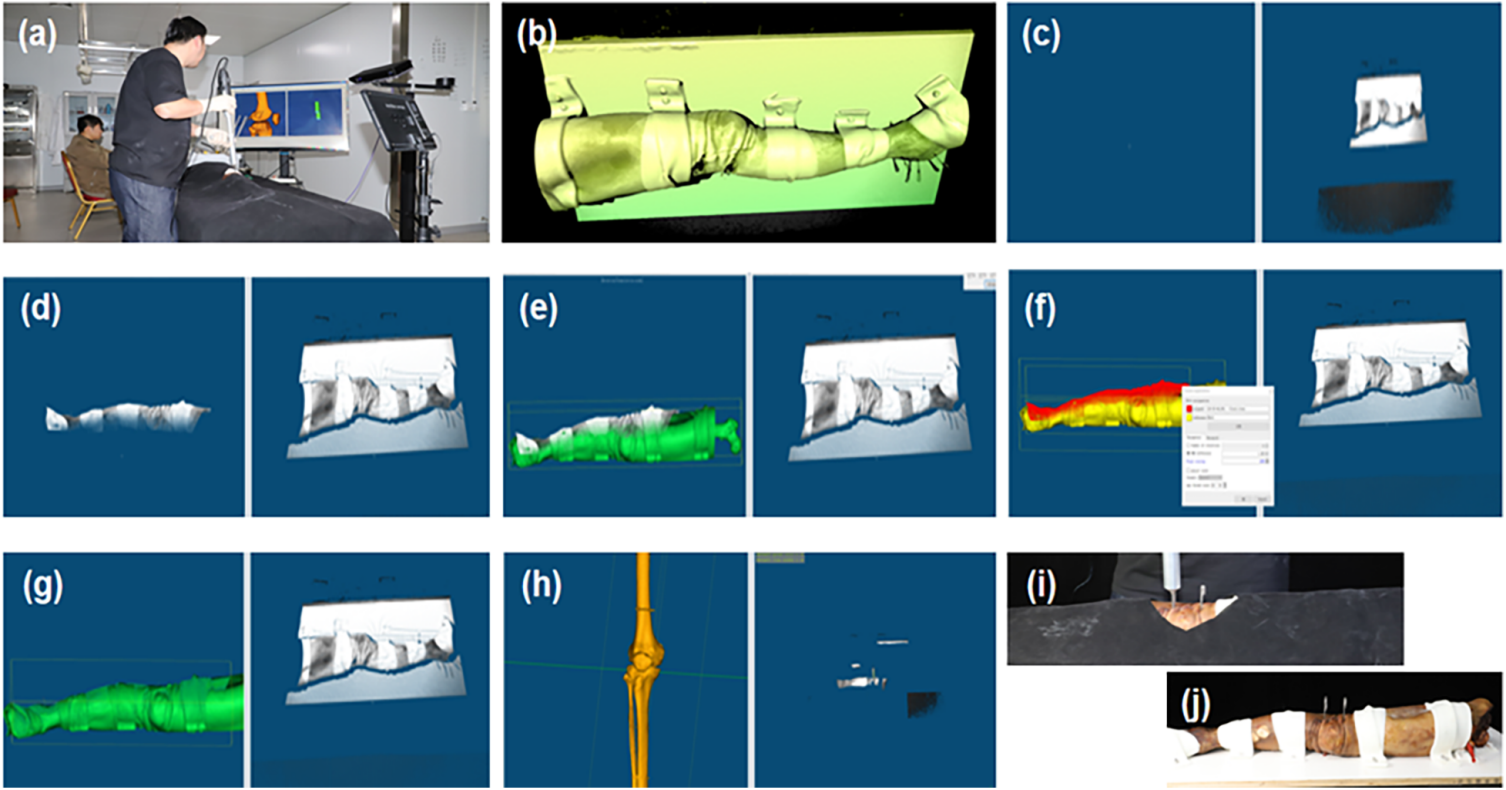
Ct and SLI guided navigation puncture. **(a)** Operation overview; **(b)** SLI preview image; **(c)** SLI image acquired; **(d)** Region of interest; **(e,f)** coarse and fine registration between the SLI surface and CT model; **(g)** intraoperative image registration results; **(h)** Post-operative bone image registration results; **(i)** Specimen puncture operations; **(j)** Post-operative puncture results.

The region of interest of the SLI point cloud for the specimen surface was cropped and denoised, and matched to the CT surface model via a coarse-to-fine image registration process. The first step is coarse alignment using multiple conspicuous surface features on the polymer bandage fixtures, and the second step obtains fine alignment by an improved speed-up version of the well established Iterative Closest Point (ICP) method (24). The quality of image registration between the SLI point cloud and the CT surface model was evaluated by a distance test function (25). The registration process was repeated until the distance was within a threshold. Following satisfactory image registration, the CT surface and skeletal models can be transformed into the SLI coordinates using (Equation 2).

(2) $$M^{SLI}=\;R*M^{\mathrm{CT}}\;+\;T$$

where $M^{\mathrm{CT}}$ represents the surface and skeleton models from CT reconstructions, and R and T the rotation matrix and translation vector as shown in (Equation 3), which are the outputs of the two-step image registration process.

(3) $$R\;=\;R^{FM}*\;R^{ICP},\;T=T^{FM}+\;T^{ICP}\;$$

where $R^{FM}$ and $T^{FM}$ are the rotation matrix and translation vector from the coarse registration by feature matching, and $R^{ICP}$ and $T^{ICP}$ the rotation matrix and translation vector from the fine registration by improved ICP. Figure 3 illustrates some snapshots of the above process.

#### 2.2.5 Real-time monitoring of puncture needle placement

**Figure 4:**
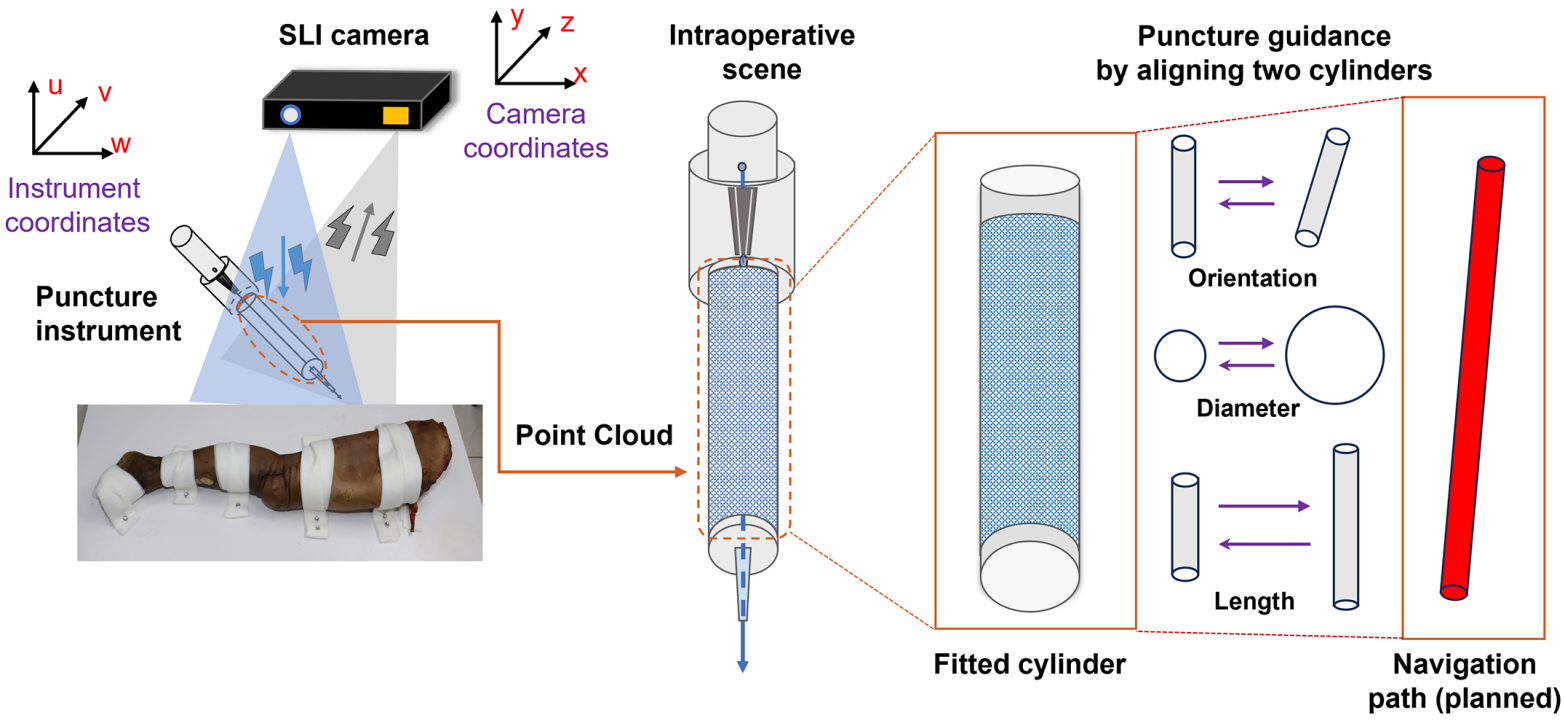
Illustration of dynamic tracking for puncture needle placement by cylinder fitting to the guiding tube SLI point cloud. Planned puncture path is also visualized as a cylinder.

Following image registration and transformation, punctuation was carried out with the help of the guiding tube. Various steps in the process and snapshots of the graphic user interface are illustrated in Figure 3. A black surgical orifice scarf was applied to the specimen surface to leave only a small area around the surgical region exposed. A point cloud of the exposed surgical area was imaged by the SLI camera (Figure 3i). The SLI camera also acquired a point cloud of the guiding tube for the puncture needle, a cylindrical model was fitted to the guiding tube point cloud to obtain its center, radius and length using the RANSAC method (26). RANSAC model fitting helps to reduce the impact of outliers in the point cloud with low computational cost. The software can visualize cylinders of different diameters and lengths to provide feedback to the surgeon, as illustrated in Figure 3a and Figure 4.

#### 2.2.6 Procedure for lower extremity skeletal puncture

Once the guiding tube was aligned with the planned path, Kirschner needles were punctured through the guiding tube in the distal femur, tibial plateau, distal tibiofibula, and sustentaculum tali positions of the lower extremity specimens. After the navigated puncture, the specimen was subjected to a spiral CT scan (with the same parameters as in preoperative scans) to assess the effect of intraoperative orthopedic puncture on skeletal drift.

### 2.3 Skin and skeletal drift assessment

**Figure 5:**
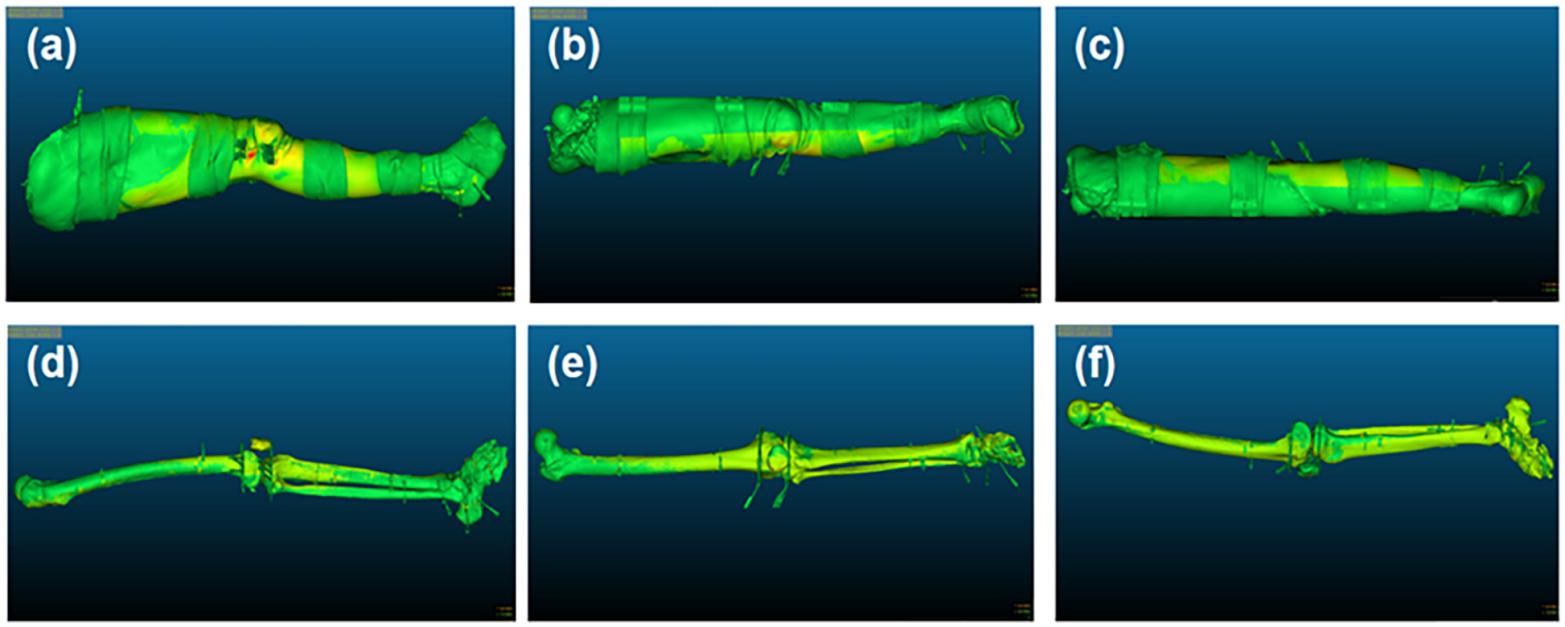
Illustration of surface and skeletal drift and mismatch. **(a–c)** The external, frontal and posterior surface of the lower limb's specimen, respectively; **(d–f)** The external, frontal and posterior surface of the specimen skeleton, respectively.

The same two-step image registration procedure as described in Section 2.2.4 was carried out. Subsequently, Root Mean Square (RMS) errors were obtained. The distance test function was used to calculate the drift and mismatch between the preoperative and postoperative specimen surfaces. The sub-matrix parameters of the preoperative specimen surface model were adjusted following image registration. This adjustment allowed for the displacement of the preoperative skeletal model to the position of the postoperative skeletal model, facilitating the visualization of the preoperative and postoperative skeletal alignment. The preoperative and postoperative skeletal drift and mismatch were calculated (Figure 5) and expressed in the form of mean ± standard deviation (M ± SD).

## 3 Results

### 3.1 Preoperative CT surface and intraoperative point cloud alignment

**Figure 6:**
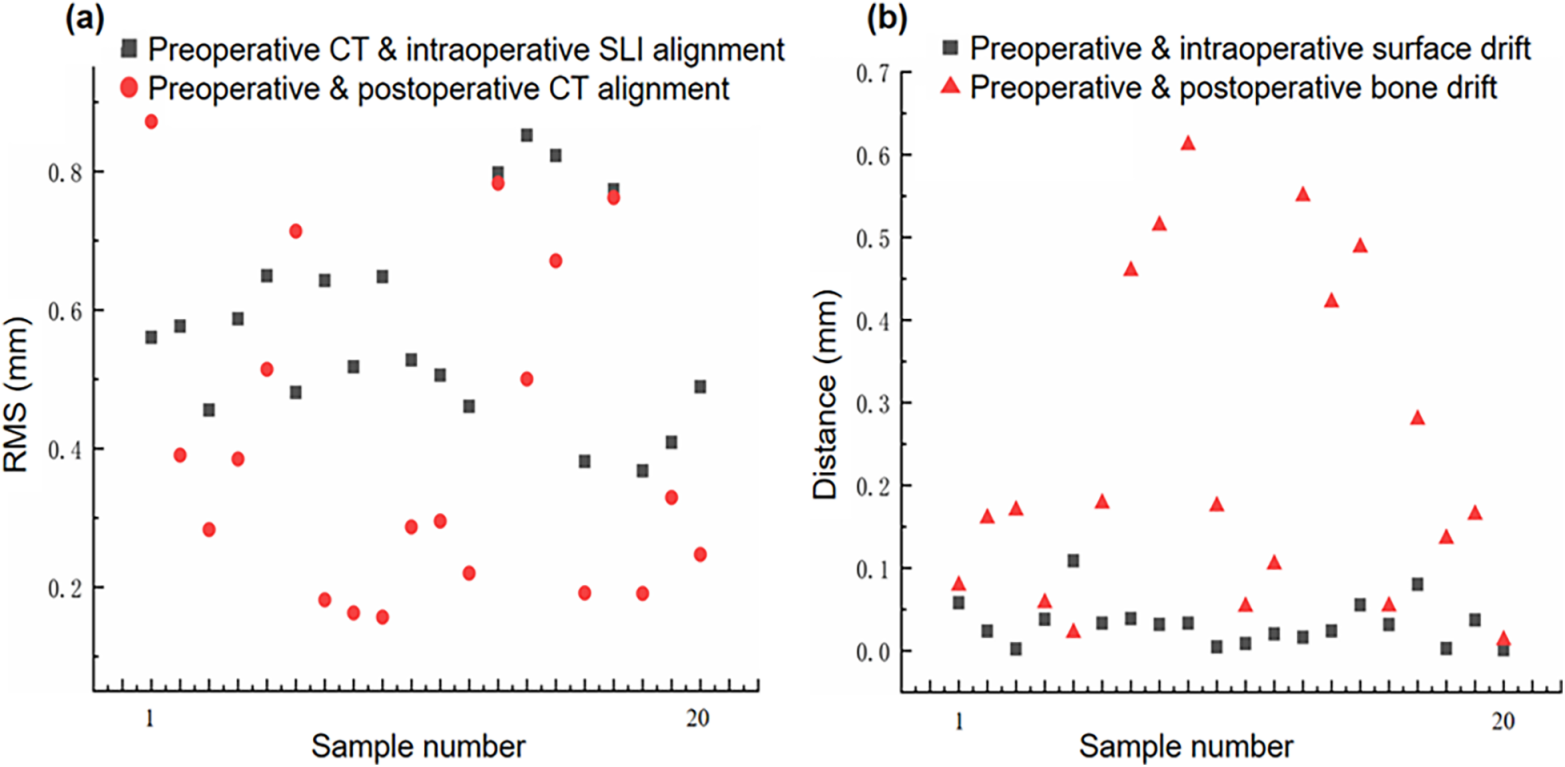
Image drift and mismatch. **(a)** Preoperative CT model vs. Intraoperative SLI surface, and preoperative and postoperative CT models alignment errors; **(b)** Preoperative and postoperative specimen surface and skeletal drift and mismatch errors.

After image registration, the RMS error between the preoperative CT surface model and intraoperative SLI surface point cloud was 0.576 ± 0.146 mm, which is statistically different from 0 (*t*-test, *p*-value < 0.05, Matlab 2023). The image acquisition methods and two-step image registration process produced satisfactory results, as depicted in Table 1 and the gray squares in Figure 6a.

**Table 1:** Results of image alignment RMS and pre-postoperative body surface and skeletal drift degrees.

	RMS errors after image alignment (mm)		Specimen surface and skeletal drift (mm)	
Specimen number	Preoperative CT vs. intraoperative SLI	Preoperative vs. postoperative CT	Surface drift	Skeletal drift
1	0.561	0.872	0.058	0.079
2	0.577	0.391	0.024	0.160
3	0.456	0.283	0.002	0.170
4	0.587	0.385	0.038	0.058
5	0.649	0.514	0.109	0.022
6	0.481	0.714	0.034	0.179
7	0.642	0.182	0.039	0.460
8	0.518	0.163	0.032	0.515
9	0.648	0.157	0.033	0.612
10	0.528	0.287	0.005	0.175
11	0.506	0.295	0.009	0.054
12	0.461	0.220	0.020	0.105
13	0.798	0.783	0.016	0.550
14	0.852	0.501	0.024	0.422
15	0.823	0.671	0.056	0.488
16	0.382	0.192	0.032	0.054
17	0.774	0.762	0.080	0.280
18	0.368	0.191	0.003	0.136
19	0.409	0.329	0.037	0.165
20	0.489	0.247	0.001	0.013
**M ± SD**	**0.576 ± 0.146**	**0.407 ± 0.234**	**0.033 ± 0.272**	**0.235 ± 0.197**

Bold values are the Means ± Standard Deviations of all samples.

### 3.2 Preoperative and postoperative CT surface model alignment

After image registration, the RMS error between the preoperative and postoperative specimen CT surface models was 0.407 ± 0.234 mm, which is statistically different from 0 (*t*-test, *p*-value < 0.05, Matlab 2023). The two models were aligned well, as shown in Table 1 and and the red circles in Figure 6a. It should be noted that preoperative and postoperative CT surface alignment error is statistically smaller than preoperative CT and intraoperative SLI surface alignment error (paired *t*-test, *p*-value < 0.05, Matlab 2023), the mean difference is −0.169 ± 0.196 mm.

### 3.3 Specimen surface drift assessment

The difference between the preoperative and postoperative body surface position was 0.0319 ± 0.0253 mm,which is statistically different from 0 (*t*-test, *p*-value < 0.05, Matlab 2023). The postoperative body surface produced a slight drift, with slight deformation of skin at the entry site in individual cadaveric specimens, as shown in Table 1 and the gray squares in Figure 6b.

### 3.4 Skeletal drift assessment

The difference between the preoperative and postoperative skeletal positions was 0.2476 ± 0.1974 mm, which is statistically different from 0 (*t*-test, *p*-value < 0.05, Matlab 2023). The postoperative skeleton produced a larger average drift than the surface, and the distribution of the skeletal drift is not as uniform as that of the surface, as shown in Table 1 and the red triangles in Figure 6b. It should be noted that preoperative and postoperative skeletal drift is statistically larger than surface drift (paired *t*-test, *p*-value < 0.05, Matlab 2023), the mean difference is 0.202 ± 0.198 mm.

## 4 Discussion

Current surgical navigation puncture systems use software or sensors to warn the surgeon or indicate possible shifts in the dynamic reference frame. Additionally, intraoperative image realignment or motion compensation algorithms are used to correct image drift and restore image mapping relationships. However, there may be a lag in the navigation system as the image refresh frequency is limited (7, 27). Some studies have reported intraoperative image drift by the image alignment error parameters (14), yet image drift includes not only intraoperative image alignment error, but also misalignment of surgical instrument mapping, and loss of reference tracking, etc.

**Figure 7:**
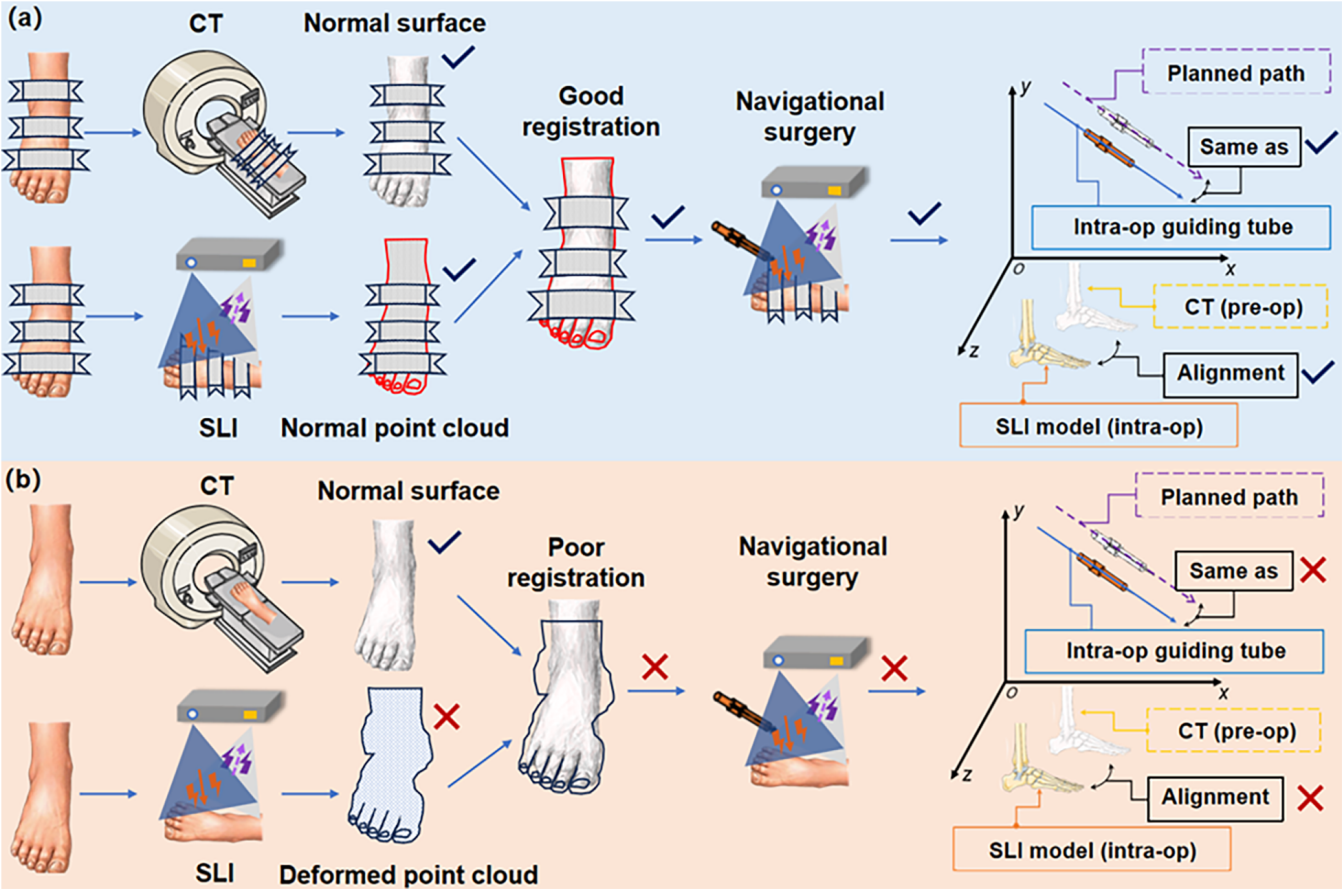
Demonstration of intraoperative image drift and mismatch sources. **(a)** Good alignment and match; **(b)** Poor alignment and match at various points in the process. (**Intra-op**: intraoperative; **pre-op**: preoperative).

In the puncture process, various operational steps and random factors can cause intraoperative image drift and mismatch, including changes in external surface morphology and internal skeletal structure, image registration between different imaging modalities, and mapping errors in surgical instruments (Figure 7). Intraoperative image drift and mismatch may accompany the entire puncture procedure, and different formats of navigation systems have different forms of intraoperative image drift. However, image spatial mapping errors affect surgical precision and can result in misplacement of guiding tubes or puncture instruments (28). Small displacements of the signal transmitter of the electromagnetic navigation system can also lead to drift in the navigation system. The distance between the receiver and the surgical site determines the degree of drift (29).

### 4.1 Error factors in image-guided orthopedic navigation puncture system

There can be a number of sources of intraoperative image drift and mismatch, as illustrated in Figure 7. These error sources are discussed below.

#### 4.1.1 Preoperative CT scan error

Preoperative CT scan image distortion can lead to intraoperative image drift and mismatch. Improper settings of CT equipment parameters can lead to reduced imaging quality, resulting in image distortions (30–33). Current clinical CT scanning often use a 512*512 image size with a pixel size of 0.5 mm. This has a minor impact on the overall imaging quality, but it might result in distortions in low-quality images (34). In addition, errors in the CT 3D reconstruction algorithm can lead to deformation of soft and skeletal tissue deformation (35, 36). A previous study using a machined part mimicking a human bone showed axial error of 0.54 mm in the cross-section, and about 70% of the error was attributed to CT scan slice thickness and image post-processing (37). Furthermore, the patient's respiration, body position, and movement produce motion artifacts throughout the scanning procedure. These artifacts have a direct impact on the precision of intraoperative image alignment.

#### 4.1.2 Intraoperative scene SLI error

Current image-guided surgical navigation systems include optical, electromagnetic, augumented reality, and inertial navigation, etc. They generally have two indicators for precision: mechanical precision and system precision. The evaluation of navigation systems mostly focuses on mechanical precision, with limited literature available on comparative assessments. Various factors during navigation surgery influence the clinician's cognition, judgment, and puncture operation. Therefore, it is important to devise solutions to help reduce navigation surgery errors caused by intraoperative image drift and mismatch. Rivkin et al. found that when the relative positions of the reference frame and the target surgical site are in close proximity, the likelihood of accidental contact between the reference system and the surgical instruments increases. Such contact may affect surgical maneuvers and image guided instrument views, or reduce navigation system precision (38).

#### 4.1.3 Alignment errors in navigation maps

Navigation system navigation map is converted into the world coordinate system. Whether based on markers, textures, anatomical landmarks, screws (39–41) or surface form (42–45) image alignment techniques, the transformation is subject to certain errors. Ommaya catheter positioning error varies significantly at the target entry point and insertion trajectory, and a single image alignment in the navigation system cannot appropriately reflect the instrument trajectory and target error, so the procedural risk needs to be carefully assessed (46). In addition, there are large differences in the acceptable range of alignment precision between different navigation systems (47).

#### 4.1.4 Tracking error of surgical instrument

Instrument tracking precision is also an important factor affecting the precision of surgical navigation procedures. Particularly in optical navigation systems, calibration of the reference frame and surgical instrument or tool is required in the intraoperative phase to determine the correct relationship between the optical tracking device and the instrument tip (7). However, after calibration, the instrument placement of optical navigation systems may still have an error of approximately 0.6 mm (48). Intraoperative image drift or even navigation failure can be caused by reference frame displacement by human error, light source projection and reflection masking (49), and spatial mapping misalignment of the instrument (50). The loss of navigation markers in a brief period of time is unlikely to affect the surgeon's operation, whereas the cumulative error of marker loss over a long period of time can lead to unintentional surgical failure.

#### 4.1.5 Image drift caused by surgical operations

The elasticity and supportive nature of soft tissues and the rigidity of bone tissues are also responsible for navigation errors. When a puncture instrument reaches a surgical site, the tissue surface may deform. The morphology of the diseased tissue may not be able to return to its initial state. For example, deformation of brain tissue may be caused by gravity, cerebrospinal fluid (CSF) leakage, tissue resection, edema, brain tissue swelling, and medication administration (51, 52). During puncture, problems such as hand drill rotation speed, cutting angle, cutting area, and poor matching of drill material to bone hardness can easily lead to insufficient drill cutting force (53–55). This phenomenon causes clinicians to apply more force to the electric hand drill, leading to an imperceptible spatial displacement of the bone.

### 4.2 Interpretation of experimental results

The experimental results showed that after the TSSF treatment, CT-SLI combined two-step alignment and guiding tube model fitting for navigation puncture, the proposed method achieved image registration RMS errors of 0.576 ± 0.146 mm and 0.407 ± 0.234 mm between preoperative CT and intraoperative SLI surface models and between preoperative and postoperative CT surface models. In addition, preoperative and postoperative specimen surface and skeletal drifts were 0.033 ± 0.272 mm and 0.235 ± 0.197 mm respectively. Though these results are statistically different from zeros, there magnitudes indicate that the proposed method can reduce and quantify intra-operative image drift and achieve the precision navigation for orthopaedic punctures.

First, the TSSF treatment enables the preservation of the body surface in the plank-fixing position without significant displacement and deformation during operations. Second, the large field of view and depth of filed of the SLI camera enables capturing surface point cloud of a long segment of the specimen such that there are vast amount of data points for the registration between CT surface model and SLI point cloud. Combined with the two-step coarse to fine image registration, this reduces image alignment errors.

In the navigation system, we aligned the preoperative CT surface model with the intraoperative SLI surface point cloud. The distance between the two is continuously monitored. We also assessed the presence of significant specimen deformations by utilizing the distance function and a heat map. In addition, the real-time surface capturing and fitting of the guiding tube offer precise alignment of the puncture instrument and the planned path,and provide feedback to surgeons. Thus, we can determine the presence of image drift and mismatch during the navigation surgery. Notably, though the preoperative and postoperative average skeletal drift was small, it was greater than surface drift (0.235 mm vs. 0.033 mm), this may be partly attributed to three factors: TSSF fixed the surface better than the bone due to the bone's surrounding soft tissue, during puncture the external force had greater impact on the bone than on the surface, and bone drift is an accumulated error that include surface drift and errors from the previous two factors.

The reduced incidence of skeletal image drift may also be related to the use of a high-speed drill during puncture. We employed a high-speed drill at 15,000 rpm/min to easily break through the bone cortex and reach the target puncture site after insertion of the needle.

### 4.3 Limitations

In the current SLI module, the XYZ resolution is mostly limited by the DMD resolution, the required large field of view and the long working distance, which are necessary for it to track a large section of the specimen as well as the surgical instrument. Given the geometrical constraints of the surgical system setup, to improve the SLI resolution it is necessary to switch to higher resolution DMDs that are much more expensive and not widely available partly due to vendor monopoly. The progress in alternative technologies, such as Liquid Crystal Display (LCD), Liquid Crystal on Silicon (LCoS) and Micro-Electro-Mechanical System (MEMS), may alleviate this issue in the near future. In addition, the SLI reconstructs one 3D image from multiple projected patterns, though it takes less than 0.5 s for each 3D image acquisition and reconstruction, it is still prone to errors due to motion. This issue can be mitigated by using a much faster SLI module, such as the MotionCam-3D^(R)^ from Photoneo (Bratislava, Slovakia), which costs about twice as the one used in the current study.

Cross-modality image registration is always challenging. The image formation mechanisms are completely different between the CT and the SLI, with different noise characteristics. As such, cross-modality registration accuracy is generally not comparable to that of same-modality registration. However, as pointed out earlier, the SLI has much higher resolution and is more robust than the binocular navigation systems based on tracking markers or light sources. In addition, the number of point clouds of the CT model is extremely large, reaching tens of millions to hundreds of millions, which affects the registration speed. With the advancement in artificial intelligence, we believe cross-modality image registration using deep learning will have higher accuracy and efficiency in the future.

Currently, simple mechanical fixtures are utilized to assist in navigation puncture, which is less flexible than multi-axis robotic arms. We are actively conducting improvements that can better take advantage of the SLI-CT navigation capability, and plan to integrate a collaborative robotic arm into the system.

In the current surgical protocol, the main 3D features used to register the SLI and CT are from the bandages and related fixtures. Polyurethane bandages quickly harden when they encounter water vapor in the air, which lead to difficulties for the disinfection work. We considering a disinfection scheme to other methods.

## 5 Conclusion

We have developed a CT and SLI image-guided orthopedic navigation puncture system in combination with three stage surface fixation and a guiding tube to reduce intraoperative image drift and mismatch, and improve surgical precision. Polymer bandages are employed to pressurize, plasticize, immobilize and toughen the surface of a specimen for surgical operations. Preoperative CT images of the specimen are acquired, a 3D navigation map is reconstructed and a puncture path planned accordingly. During surgery, an SLI module captures and reconstructs the 3D surfaces of both the specimen and a guiding tube for the puncture needle. The proposed system has been tested and evaluated using 20 formalin-soaked lower limb cadaver specimens. The results indicate the proposed method is effective in reducing intraoperative image drift and mismatch.

## Data Availability

The raw data supporting the conclusions of this article will be made available by the authors, without undue reservation.
